# State-Level Affordability of Factory-Made Cigarettes among Current US Smokers: Findings from the ITC US Survey, 2003–2015

**DOI:** 10.3390/ijerph16132439

**Published:** 2019-07-09

**Authors:** Pete Driezen, Nigar Nargis, Mary E. Thompson, K. Michael Cummings, Geoffrey T. Fong, Frank J. Chaloupka, Ce Shang, Kai-Wen Cheng

**Affiliations:** 1 Department of Psychology, University of Waterloo, Waterloo, ON N2L 3G1, Canada; 2 School of Public Health and Health Systems, University of Waterloo, Waterloo, ON N2L 3G1, Canada; 3 Economic and Health Policy Research, American Cancer Society, Washington, DC 20004, USA; 4 Department of Statistics and Actuarial Science, University of Waterloo, Waterloo, ON N2L 3G1, Canada; 5 Department of Psychiatry and Behavioral Sciences, Medical University of South Carolina, Charleston, SC 29425, USA; 6 Ontario Institute for Cancer Research, Toronto, ON M5G 0A3, Canada; 7 Division of Health Policy and Administration, School of Public Health, University of Illinois at Chicago, Chicago, IL 60612-4394, USA; 8 National Bureau of Economic Research, Cambridge, MA 02138, USA; 9 Oklahoma Tobacco Research Center, Stephenson Cancer Center, The University of Oklahoma Health Sciences Center, Oklahoma, OK 73104, USA; 10 Department of Health Administration, Governors State University, University Park, IL 60484-0975, USA; 11 Institute for Health Research and Policy, University of Illinois at Chicago, Chicago, IL 60612-4394, USA

**Keywords:** United States, tobacco, economics, price, taxation, affordability, small area estimation

## Abstract

Cigarette affordability measures the price smokers pay for cigarettes in relation to their incomes. Affordability can be measured using the relative income price of cigarettes (RIP), or the price smokers pay to purchase 100 packs of 20 cigarettes divided by their per capita household income. Using longitudinal data from 7046 smokers participating in the International Tobacco Control (ITC) US Survey, the purpose of this study was to test whether affordability significantly changed following the US federal tax increase implemented on 1 April 2009. This study also estimated temporal trends in affordability from 2003–2015 at state and national levels using small area estimation methods and segmented linear mixed effects regression models. RIP increased slightly during 2003–2008. This was followed by a 30% increase during 2008–2010, indicating cigarettes were less affordable after the federal tax increase. RIP continued to increase during 2010–2013 but decreased during 2013–2015, suggesting cigarettes have recently become more affordable for US smokers. State-level trends in RIP were consistent with overall national trends. Controlling for other factors, a $1 increase in the state excise tax was significantly associated with a 9% increase in RIP, indicating state taxes reduced affordability. Tax-induced price increases must keep pace with underlying economic conditions to ensure cigarettes do not become more affordable over time.

## 1. Introduction

The price of tobacco products influences consumer behavior. In high income countries, a 10% increase in the price of tobacco reduces consumption by 4%, usually by encouraging smokers to quit, reducing consumption among continuing smokers, and preventing youth from starting to smoke [1,2,3]. Governments directly influence prices by levying excise taxes on tobacco products. Tobacco taxation is recognized as the most effective tobacco control policy available to governments to reduce the prevalence of tobacco use [1,2,3].

In the United States, excise taxes are levied on tobacco products at federal, state, and local levels. Both federal and state excise taxes are set legislatively and are collected before the point of sale. From 2 January 2002 to 1 April 2009, the federal excise tax on cigarettes was $0.39/pack. This was increased to $1.01/pack on 1 April 2009 [4,5]. At that time, South Carolina had the lowest state-level cigarette excise tax ($0.07/pack) while New York had the highest ($2.75/pack). On average, major tobacco growing states generally had lower taxes ($0.385/pack) compared to all other states [5] ($1.31/pack). By 2015, New York levied the highest tax ($4.35/pack), followed by Rhode Island ($3.75/pack), Connecticut ($3.65/pack), Massachusetts ($3.51/pack), and Hawaii ($3.20/pack) [6]. Several local jurisdictions also tax cigarettes, including New York City; Chicago; Cook County, Illinois; and Philadelphia, Pennsylvania [7].

Although tobacco excise tax policy directly influences the price of cigarettes, the beneficial effects of increased prices can erode over time in the presence of rapid economic growth. Under such conditions, smokers’ incomes may increase faster than cigarette prices, making cigarettes more affordable [8,9,10,11,12]. It is now recognized that cigarette affordability, or the price of cigarettes in relation to a smoker’s income, influences purchasing behavior and, ultimately, cigarette consumption and smoking prevalence [11,12,13].

Early studies of cigarette affordability relied on aggregate measures of affordability, either defined as the minutes of labor for a typical wage earner to purchase one standard pack of cigarettes (i.e., Marlboro) or the relative income price of cigarettes, defined as the percentage of per capita gross domestic product needed to purchase 100 standard packs of cigarettes [13,14]. While cigarettes generally cost more in high-income countries compared to low- and middle-income countries (LMICs), they are typically more affordable in high-income countries [8,15]. In the early 2000s, cigarettes became more affordable in LMICs, particularly in countries experiencing rapid economic growth [8,13]. Blecher and van Walbeek [13] further noted that cigarettes became less affordable in high income countries where rapid tax increases were a central part of focused tobacco control policies.

In the United States, Bandi et al. [16] examined temporal and geographic trends in aggregate measures of cigarette affordability across all US states. At a national level, cigarettes became more affordable from 1970 to 1978, less affordable from 1978 to 2001, and slightly more affordable from 2001 to 2008. This slight increase in affordability was attributable to relatively stagnant cigarette prices and rising incomes [16]. By 2010, cigarettes were least affordable in northeastern US states and most affordable in southeastern states and parts of the West [16].

While aggregate estimates of cigarette affordability illustrate how differences in price and economic conditions influence affordability, they are unable to capture how individual-level factors influence affordability. In a study of the financial burden of purchasing cigarettes in New York City, Farrelly et al. estimated the share of household income spent on cigarettes by different income groups using data from the New York Adult Tobacco Survey [17]. In 2003/2004, low income smokers earning less than $30,000/year spent 11.6% of their household income on cigarettes. By 2010/2011, low-income smokers spent almost twice as much on cigarettes (23.6% of household income). Meanwhile, high-income smokers earning $60,000/year or greater only spent 2% of their household income on cigarettes in each survey year.

Nargis et al. [11] adapted the relative income price metric to study individual-level affordability of low-, medium-, high-, and premium-price cigarette brands in Bangladesh from 2009 to 2015. Using self-reported prices from four waves of the International Tobacco Control (ITC) Bangladesh Survey, they defined relative income price as the price required to purchase 100 standard packs of 20 cigarettes divided by annual per capita household income. While low-priced brands became slightly less affordable, medium-, high-, and premium-price brands became more affordable. This increased affordability coincided with a period of rapid growth in household income [11].

Using relative income price, another study from China showed that cigarettes became significantly more affordable for Chinese smokers beginning in 2007. This trend continued until 2015, in spite of a 2009 tax increase [12]. Nargis et al. [12] concluded that growth in disposable incomes among Chinese smokers contributed to increased cigarette affordability and consumption. Not only did affordability increase over time, it also varied by sociodemographic factors. In particular, cigarettes were more affordable among more educated smokers, older smokers, and smokers of high socioeconomic status [11,12].

Because of the strong and established causal relationship between affordability and subsequent smoking [1,10,12], it is important to examine the factors that influence cigarette affordability. There are two main factors: (1) the actions of producers and retailers on the price of cigarettes; and (2) the taxes that governments impose on cigarettes. Because of the need to understand the impact of policies on addressing the number one preventable cause of death—smoking—this study examined the impact of cigarette taxes on affordability. Using individual-level data from the US arm of the ITC Four Country Survey, the primary purpose of this study was to test whether affordability significantly changed following the 1 April 2009 federal cigarette excise tax increase. Given the unique longitudinal data available, this study also examined whether prevailing economic conditions influenced cigarette affordability during the US recession of 2008–2009 and in subsequent years. Specifically, wave-to-wave changes in affordability were estimated following the federal tax increase to test the effects of improved economic conditions following the recession. Finally, this study examined whether sociodemographic characteristics and state-level taxes influenced affordability over and above prevailing economic conditions.

## 2. Materials and Methods

### 2.1. Data Sources

This study relied on three separate data sources to estimate individual-level cigarette affordability across all US states (Table 1). The primary data came from the ITC US Survey. Contemporaneous data from the American Community Survey (ACS) were used to impute a more granular measure of household income for all ITC US respondents. Finally, state-level auxiliary estimates from the US Behavioral Risk Factor Surveillance System (BRFSS) were used to reliably estimate affordability across all US states.

#### 2.1.1. The ITC US Survey

The ITC US Survey was a prospective cohort study of smokers conducted from 2002 (Wave 1) to 2015 (Wave 9). In the first wave of the ITC Survey, a nationally representative sample of 2000 adult smokers aged 18 and older was selected from 14 geographic strata in the United States using a stratified probability-based sampling design [18]. Geographic strata were comprised of either single states or groups of states. Respondents were interviewed using computer-assisted telephone interviewing (CATI) in the first six waves, while CATI and computer-assisted web interviewing were used for Waves 7–9. Smokers lost to attrition were replaced with new respondents sampled using the same design. Respondents who quit smoking were also followed over time. Approximately 2000 current or former smokers were interviewed per wave in each of the first six waves. In Waves 7 and 8, respectively, the sample was reduced to about 1750 and 1500 respondents while, in Wave 9, about 3200 current and former smokers were interviewed. All respondents provided informed consent to participate in the ITC Survey. The survey protocols and all materials, including the survey questionnaires, were cleared for ethics by the Office of Research Ethics, University of Waterloo, Canada (ORE #13978 and ORE #17469) and the Institutional Review Board (NT 02-20) at Roswell Park Cancer Center, Buffalo, New York, USA. Additional details of the study are available elsewhere [18,19].

The current study was based on 7046 current smokers of factory-made cigarettes who participated in at least one wave of the ITC US survey from 2003 (Wave 2) to 2015 (Wave 9). These smokers contributed 13,191 observations to the analysis reported here. Data from Wave 1 were excluded from this study because information on the number of children living in a smoker’s household was not collected in that wave. This information was needed to impute per capita household income from the American Community Survey. ITC data from Waves 2–8 were treated as one time point per wave while data from Wave 9 were split into two sub-waves. This was done because re-contact respondents in Wave 9 were primarily surveyed in 2013 and 2014. Telephone recruitment of new respondents was also attempted during this time. Due to difficulties in recruiting new respondents by telephone, the majority of new respondents were recruited from GfK’s web-based Knowledge Panel in 2015 [20].

#### 2.1.2. The American Community Survey

The US Census Bureau administers the American Community Survey. It collects data on a monthly basis from approximately 3.5 million household addresses each year. The series of monthly samples provide reliable annual estimates of key demographic, housing, social, and economic indicators [21,22]. Data from ACS annual Public Use Microdata Sample files were obtained from the US Census Bureau’s website [23]. Household income for ITC respondents was imputed from the contemporaneous ACS; thus, if an ITC survey spanned two calendar years, ACS data from the same calendar years were used to impute household income.

#### 2.1.3. The Behavioral Risk Factor Surveillance System

The US BRFSS is the largest health survey in the world, interviewing more than 400,000 respondents each year [24,25]. Public use microdata files were obtained directly from the US Centers for Disease Control and Prevention website [26]. These data were used to estimate state-level proportions of sex, age group, race, education, and employment status among current smokers. BRFSS data were also used to estimate the total number of cigarette smokers in each state across all years in which an ITC survey was conducted. These aggregate estimates were then used as auxiliary information in small area estimation models described below.

### 2.2. Measures

#### 2.2.1. Imputed Household Income

The ITC US Survey collected household income information using eight broad categories. To estimate cigarette affordability, a granular proxy measure of annual per capita household income was required. This measure was imputed for all ITC respondents using information from the American Community Survey (Figure S1) by adapting a procedure from Nargis et al. [11]. Annual per capita incomes were imputed according to survey year, state of residence, broad income category, and the ages of children living in a respondent’s home (children under 6, children aged 6–17, children in both age groups, or no children in the home). Each of these measures was identically defined in both the ITC and ACS datasets, with the exception of the Wave 8 and Wave 9 ITC Surveys. For these years, the contemporaneous ACS survey used a different definition of children in the home: children under 5 and children aged 5–17. Given the age ranges differ by only one year and that per capita income imputation was conducted within year, the impact on per capita income was negligible.

For the ACS microdata, the continuous measure of household income was classified into the eight categories used in the ITC survey. The age of children living in an ACS respondent’s home was also classified into comparable categories as in the ITC survey. Per capita income from the ACS was then computed as reported annual household income divided by the total number of people living in that household. At this point, mean per capita household income was estimated (suitably weighted using the ACS sampling weights) within 32 income-by-child groups for all states for each calendar year in which an ITC survey was conducted. These mean estimates were then assigned to all ITC respondents within the same calendar year, US state, income, and child group, yielding refined estimates of per capita household income for all respondents. Overall, the distribution of imputed per capita household income was relatively similar across all waves (Figure S2).

#### 2.2.2. Cigarette Affordability

The primary outcome measure for this study was cigarette affordability, defined as the relative income price (RIP) of factory-made cigarettes [11]. This measure compares the self-reported price of cigarettes to an estimate of a smoker’s annual per capita household income. Self-reported prices paid per pack of 20 cigarettes were computed for all smokers of factory-made cigarettes participating in a least one wave of the ITC US Survey. Self-reported prices were based on smokers’ last purchase, using either the total price paid for all cartons purchased or for all packs purchased divided by the number of cigarettes purchased. Unit prices were converted to price per pack of 20 cigarettes by multiplying the unit price by 20. RIP was then defined as the self-reported price needed to purchase 100 standard packs of 20 cigarettes divided by a smoker’s annual per capita household income. Higher values of RIP indicate lower cigarette affordability, meaning that smokers spend a larger share of their household income on cigarettes.

#### 2.2.3. Covariates

Several individual-level measures from the ITC surveys were used as covariates in both wave-specific small area estimation models and in linear mixed effects models estimated to examine temporal trends in RIP. Individual-level covariates included sex (female vs. male), age group (25–39, 40–54, 55+ vs. 18–24), race/ethnicity (black, Hispanic, other vs. white), education (high school or less vs. greater), and employment status (employed vs. otherwise).

Longitudinal linear mixed effects models included two additional state-level covariates: state-level excise taxes adjusted for inflation to 2015 US dollars and the annual state-level labor force participation rate, measured in percentage points. State-level excise tax data ($/pack) were obtained from the STATE system maintained by the CDC [6] while labor force participation rates were obtained from US Bureau of Labor Force Statistics [27]. Excise tax rates were added to the ITC data according to the state in which ITC respondents lived and the calendar quarter in which they were surveyed. Labor force participation rates were added according to the state in which ITC respondents lived and the calendar year in which they were surveyed.

#### 2.2.4. Auxiliary Measures

Unit-level small area estimation models were used to estimate wave-specific individual-level cigarette affordability across all US states. A unique feature of these models is that they employ auxiliary information to reliably estimate quantities of interest in survey domains (or “small areas”) having small sample sizes. Since the ITC US Survey primarily used groups of states as the sampling strata, states having small populations generally had small sample sizes (typically fewer than 50 smokers per wave). Estimates for these individual states therefore exhibit greater variability than states having larger populations (and therefore larger sample sizes in the ITC US Survey, e.g., New York, California).

Auxiliary measures consisted of state-specific population averages of sociodemographic characteristics potentially related to RIP, including sex, age, race/ethnicity, education, and employment status [11]. All auxiliary measures were estimated using current smokers from the BRFSS Survey contemporaneous to each ITC wave (Table 1). Thus, auxiliary measures were the state-level proportion of current smokers (suitably weighted) who were:

- female;
- age 25–39;
- age 40–54;
- age 55+;
- black;
- Hispanic;
- from other racial groups;
- had a high school education or less; and
- employed.

In addition, the total number of people who were current smokers in each state was also estimated using BRFSS data.

### 2.3. Statistical Analysis

#### 2.3.1. Small Area Estimation Models

It is often useful to estimate statistics in population subgroups for which a particular survey was not designed [28,29]. The samples from these subgroups are typically small; as a result, direct subgroup estimates are statistically unreliable. Such subgroups or “domains” may represent minority groups in the population or small geographic areas. The need for reliable estimates in these small domains has led to the development of statistical small area estimation methods which link domains through common parameters to “borrow strength” from related domains and improve estimation efficiency. Small area estimation models typically estimate small domain statistics using mixed effects regression models, defined as

(1) $$Y_{is}=x_{is}^{⊤}\beta +v_{s}+e_{is}$$

where $Y_{is}$ is a continuous outcome measure for respondent *i* in area *s*, $x_{is}$ is a vector of covariates for respondent *i* in area *s*, and β is a vector of estimated regression parameters. Random area effects are represented by $v_{s}$, which are independent and identically distributed as $N(0,\sigma_{v}^{2})$ and $e_{is}$ are individual errors also independent and identically distributed as $N(0,\sigma_{e}^{2})$. Note that the random area effects $v_{s}$ are typically treated as random intercepts in these models.

In this study, small area estimation models were used to estimate statistics of self-reported cigarette prices per pack (inflation adjusted to 2015 USD) and RIP across all US states over time. Since the geographic sampling strata in the ITC US Survey were primarily comprised of groups of US states, there were typically fewer than 50 smokers in any given state for any wave of the ITC US Survey. The exceptions were states having large populations (NY, PA, IL, MI, OH, FL, TX, and CA). RIP was therefore estimated using a linear mixed effects (LME) regression model [29,30]. This particular model relates individual-level RIP to individual-level fixed-effect covariates (sex, age group, race/ethnicity, education, and employment status). State-level effects are incorporated into the model as random intercepts. Model estimates are then combined with external, auxiliary information to estimate the empirical best linear unbiased predictors (EBLUPs) of RIP for each state within each wave of the ITC US Survey. Specifically, EBLUPs are estimated using the auxiliary information, fixed effect parameter estimates and the random intercept for each state (see Molina and Marhuenda [29] for computational details). For estimation purposes, these same measures must be included as fixed effects in the LME model.

A separate LME model was estimated for each of Waves 2–9 of the ITC US Survey. Again, data for Wave 9 were split into two sub-waves, yielding nine sets of affordability estimates for all US states. Regression diagnostics of initial models suggested that model residuals were positively skewed. Thus, a log transformation of RIP was used to estimate cigarette affordability. EBLUP estimates of log(RIP) were back-transformed to produce geometric means of the RIP of factory-made cigarettes. State-specific estimates were then mapped over all waves to visualize changes in RIP over time. All analysis was conducted using the R statistical software package (Version 3.4.3). Small area estimation models were estimated using the “sae” package [29] (Version 1.1).

#### 2.3.2. Longitudinal Modeling of Temporal Trends

Linear mixed effects models were also used to test changes in RIP over time. A segmented regression model fit specific piecewise trends in log(RIP) based on the timing of the 2009 federal tax increase as well as the timing of the US recession and subsequent economic recovery. Temporal effects were incorporated into each of the models using survey wave as a proxy measure for time. Models also allowed for repeated measurements made on respondents over multiple *t* time points. Thus, longitudinal LME models were of the general form

(2) $$\begin{array}{cc} \log{\left(\mathrm{RIP}\right)}_{tis}= & \beta_{0}+{x_{\mathrm{DEM}}}_{tis}^{⊤}\beta_{\mathrm{DEM}}+\beta W7_{tis}+\beta TAX_{tis}+\\ & \beta LF_{tis}+{x_{\mathrm{PERIOD}}}_{tis}^{⊤}\beta_{\mathrm{PERIOD}}+u_{is}+v_{s}+e_{tis} \end{array}$$

In this general form, ${x_{\mathrm{DEM}}}_{tis}$ is a vector of sociodemographic indicator variables (female, age group, race, education, and employment status) for respondent *i* at time *t* in state *s*. All sociodemographic variables were entered as fixed effects in these LME models. Additional fixed effects were:

- a dummy indicator W7 for smokers surveyed in Wave 7 after 1 April 2009 to control for possible differences in log(RIP) in this group;
- a TAX variable to represent state-level cigarette excise taxes adjusted for inflation to 2015 USD; and
- a measure, LF, of the state-level labor force participation rate to account for varying economic conditions between states and over time.

Different piecewise trends in log(RIP) were estimated using four separate longitudinal LME models. Thus, the general term ${x_{\mathrm{PERIOD}}}_{tis}$ defined one or more period covariates in each of the four models:

- Model 1: a single slope model that estimated $\beta_{\mathrm{period}\;1}$ representing the wave-to-wave linear trend in log(RIP) over the entire study period;
- Model 2: a two-slope model that estimated a linear trend in log(RIP) from 2003 to 2008 ($\beta_{\mathrm{period}\;1}$) and a second linear trend from 2008 to 2015 ($\beta_{\mathrm{period}\;2}$);
- Model 3: a three-slope model that estimated a linear trend in log(RIP) from 2003 to 2008 ($\beta_{\mathrm{period}\;1}$), a linear trend from 2008 to 2010 ($\beta_{\mathrm{period}\;2}$), and a final linear trend from 2010 to 2015 ($\beta_{\mathrm{period}\;3}$); and
- Model 4: a four-slope model that estimated the first two linear trends from Model 3 as well as the linear trend from 2010 to 2013 ($\beta_{\mathrm{period}\;3}$) and the linear trend from 2013 to 2015 ($\beta_{\mathrm{period}\;4}$).

By way of example, Model 4 estimates the following trends in log(RIP) over the course of the study:

- The first slope (“Period 1”) estimates the linear wave-to-wave change in log(RIP) from 2003 (Wave 2) to 2008–2009 (Wave 7) prior to the federal tax increase.
- The second slope (“Period 2”) estimates the linear change in log(RIP) from 2008–2009 (Wave 7) to 2010–2011 (Wave 8). This period spans the federal tax increase, the official end of the recession (June 2009), and a time of stagnant economic conditions characterized by high unemployment and reduced household income [31,32,33].
- The third slope (“Period 3”) estimates the linear trend in log(RIP) from 2010–2011 (Wave 8) to 2013–2014 (Wave 9a), a time characterized by slow economic recovery including decreasing unemployment rates and increasing household incomes [32,33].
- The final slope (“Period 4”) estimates the linear trend in log(RIP) from 2013–2014 (Wave 9a) to 2015 (Wave 9b). By 2015, key economic indicators had returned to pre-recession levels [31,32,33].

Piecewise trends were entered as fixed effects in all LME models. The random effects $u_{is}$ and $v_{s}$ were used to represent random effects for all *i* respondents measured at multiple time points within state *s* and random state effects *s*, respectively. These effects were entered as random intercepts in the models (specified as “nested” random effects). All segmented LME models were estimated in R using the “lme4” package [34] (Version 1.1-15) and the fit of each model was compared using Akaike’s Information Criteria (AIC) statistic.

## 3. Results

### 3.1. Sample Characteristics

Of 7046 current smokers of factory-made cigarettes who participated in at least one survey wave over the course of the study, 46% were men (Table 2). A smaller percentage of smokers recruited prior to Wave 7 were men compared with at least 50% of smokers recruited in Wave 7 or thereafter. The age distribution of smokers also varied by wave of recruitment such that a greater percentage of smokers recruited since Wave 7 were aged 55 or older while larger percentages of smokers recruited in earlier waves were younger than 40. The percentage of smokers having a high school education or less also varied by wave of recruitment, ranging from 37% of smokers in Wave 2 to 52% of smokers in Wave 5. Household incomes were more similar across all waves. About two-thirds of smokers did not have any children living with them while about 20% of smokers reported having at least one child aged 6–17 living in their homes. The majority of smokers were white and smoked on a daily basis, consuming, on average, 17 cigarettes per day.

### 3.2. Self-Reported Pack Prices by State

Wave-specific LME models were used to estimate the real (2015 USD), self-reported price per pack of 20 cigarettes across all waves (Table S1). As expected, there was a significant random effect of state, indicating important variation between states in cigarette prices. The intraclass correlation coefficient (ICC) ranged from 0.217 to 0.345 across all models, indicating that 22–34% of the variation in self-reported pack prices is attributable to smokers’ state of residence. This variation is depicted cartographically in Figure S3 while numeric estimates and temporal trends are displayed in Table S2. Across all time points, self-reported pack prices were higher, on average, in northeastern US states and lower in southern states. In 2003, for example, smokers from the northeast reported paying an average price of $4.78/pack. In comparison, smokers from the south reported paying an average price of $3.48/pack. Generally, smokers from all states reported paying higher prices over time, with a noticeable increase in self-reported prices beginning in 2010 following the federal excise tax increase of 2009.

### 3.3. Relative Income Price by State

While inflation-adjusted self-reported prices generally increased over the study period, personal incomes and other economic factors influence whether higher cigarette prices translate into reduced cigarette affordability. Again, wave-specific small area estimation models were used to estimate log(RIP) across all US states (Table S3). Compared to models of self-reported prices, LME model results indicate substantially smaller variation in log(RIP) between states. While the random effect of state was significant in most models, the ICC ranged from 0.013 to 0.057 across models, suggesting that only 1.3–5.7% of the variation in log(RIP) is attributable to smokers’ state of residence. Thus, even though self-reported prices vary across US states, cigarette affordability is more similar across US states, suggesting that, after personal incomes are considered, smokers living in higher-priced states have similar ability to pay for cigarettes compared to smokers living in lower-priced states. For example, average RIP (i.e., the geometric mean) in Rhode Island was 2.17 in 2003, similar to the average RIP in Georgia (2.19), Kentucky (2.21), Iowa (2.16), South Dakota (2.19), and Idaho (2.28). In other words, smokers from each of these states spent, on average, about 2.2% of their annual per capita household income on 100 packs of cigarettes. As shown in Figure S4, average RIP was more similar across all US states in any given year compared to cigarette prices (Figure S3). Indeed, average RIP showed little variation across the United States in 2007–2008 and in 2008–2009. In both of these years, the random effect of state was not statistically significant in the small area estimation models (Table S3).

State-specific estimates of RIP over time further suggest that the cigarette affordability remained relatively stable across most US states from 2003 until 2007–2008 (Table S4). After this time, RIP increased until 2013–2014. By 2015, RIP decreased in most US states. In the northeast, RIP increased from an average of 2.43 at the beginning of the US recession (2007–2008), to 3.48 in 2010–2011, well after the federal cigarette excise tax increase and the official end of the recession. Similar effects were observed in other regions during this time period. Average RIP peaked in all regions in 2013–2014 (northeast = 4.27, south = 4.53, midwest = 3.96, west = 4.54) and fell by 2015 (northeast = 3.81, south = 4.14, midwest = 3.77, and west = 3.84). While the effects of the federal tax increase on the relative income price of cigarettes cannot be disentangled from the effects of the recession based on these estimates alone, these estimates demonstrate that cigarette affordability is clearly tied to prevailing economic conditions. In other words, during the slow economic recovery that followed the official end of the US recession in June 2009, smokers of factory-made cigarettes clearly spent a larger percentage of their per capita annual household incomes on cigarettes.

### 3.4. Temporal Trends in Relative Income Price

A segmented linear mixed effects regression model was then fit to the longitudinal data to model temporal trends in log(RIP) over the entire study period. The same covariates were used as fixed effects in these models for consistency with the small area LME models used to produce state-specific estimates of log(RIP). Additional covariates were an indicator for respondents interviewed in Wave 7 following the federal tax increase and state-level fixed effects for inflation adjusted excise taxes and labor force participation rates. Two random intercepts (for state and respondent) were also included. As mentioned, four different models were estimated specifying different temporal trends (Table 3). All models were based on 6660 respondents who did not move between states during the study period. These 6660 respondents contributed 12,332 observations to the analysis.

Across all four models, there were significant random effects of both state ($\tau_{00}$, state; Table 3) and respondent ($\tau_{00}$, state:respondent; Table 3; all *p* < 0.001). A greater proportion of the variation in log(RIP) over the study period was attributable to individual smokers than state, suggesting that the relative income price of cigarettes varies less within smokers over time than between smokers, and that the state random effects vary less than the individual random effects. ICC estimates for respondent and state were similar across all models irrespective of the type of temporal trend that was estimated.

Based on model fit AIC statistics, the four-slope model was the best fitting model followed by the two-slope model. In the four-slope model, the regression coefficient for Period 1 (2003–2008, $\beta_{\mathrm{period}\;1}$) describes the wave-to-wave change in log(RIP) from Wave 2 to Wave 7 while the coefficient for Period 2 (2008–2010) describes the difference in the slope from Period 1 to Period 2. As a result, the slope from Period 1 to Period 2 is estimated as $\beta_{\mathrm{period}\;1}+\beta_{\mathrm{period}\;2}$. Using this interpretation, the wave-to-wave rate of change in log(RIP) from 2003 to 2008 was 0.034 (*p* < 0.001), while the change from 2008 to 2010 was 0.034 + 0.227 = 0.261. Since log(RIP) was used as the outcome variable, the percent change in RIP for each time period is estimated as

(3) $$\begin{array}{c} {}[\exp\left(\beta_{\mathrm{period}\;1}\right)-1]\times 100,\;\mathrm{and}\\ \exp(\beta_{\mathrm{period}\;1}+\beta_{\mathrm{period}\;2})-1]\times 100 \end{array}$$

**Figure 1:**
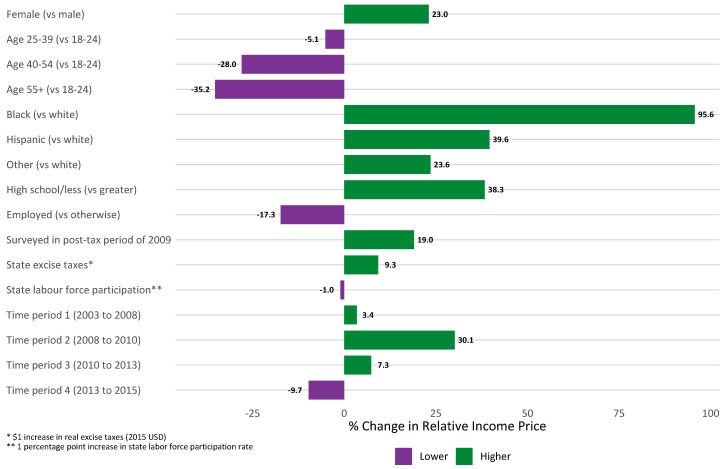
Percent change in RIP among US smokers from 2003 to 2015 (longitudinal LME regression, *n* = 6660).

Thus, there was a 3.4% wave-to-wave increase in the relative income price of cigarettes from 2003 to 2008 (Wave 2 to Wave 7) followed by a 30.1% increase in RIP from 2008 to 2010 (Wave 7 to Wave 8). Put another way, factory-made cigarettes became slightly less affordable across the United States from 2003 to 2008. Following the federal excise tax increase and economic recession, cigarettes became 30% less affordable (Figure 1). RIP increased by 7.3% [(exp(0.033 + 0.230 − 0.192) − 1) × 100] from 2010 to 2013 and then decreased by 9.7% [(exp(0.033 + 0.230 − 0.192 − 0.173) − 1) × 100] from 2013 to 2015. These effects suggest that cigarettes continued to be less affordable from 2010 to 2013. By 2015, following complete economic recovery, factory-made cigarettes were becoming more affordable for American smokers, although they were less affordable at this time than they were prior to the economic recession and federal excise tax increase.

At a national level, the four-slope model appears to capture the general trend in cigarette affordability depicted by the state-specific temporal trends in Table S4. The “Trend” column of that table demonstrates that cigarette RIP peaked in most states around 2013 (corresponding to the first half of the ITC US Wave 9 survey). Following that time, RIP decreased in 38 states, meaning that cigarettes became more affordable in most areas throughout the United States from 2013 to 2015.

Fixed effect parameter estimates for sociodemographic covariates were similar across all models regardless of how the temporal trend was modeled. Based on the four-slope model (Table 3, Figure 1), over the course of the entire study, female smokers had 23% higher RIP than male smokers, meaning that female smokers tended to spend a greater share of their annual per capita household incomes on cigarettes compared to male smokers even after controlling for other factors. On the other hand, the oldest smokers (age 55+) spent a significantly smaller proportion of their annual per capita household incomes on cigarettes compared to the youngest smokers. A similar effect was found for employed smokers, who spent 17% less on cigarettes than smokers not currently employed. Smokers from all other racial groups spent a significantly greater percentage of their annual per capita household incomes on cigarettes than white smokers (all *p* < 0.001), with black smokers spending 96% more on cigarettes than white smokers. Interestingly, the small fraction of smokers interviewed in Wave 7 immediately following the federal tax increase had significantly higher RIP than other smokers, suggesting the tax increase, at least in part, reduced cigarette affordability in this group of smokers.

State-level fixed effects were also associated with the relative income price of cigarettes. In particular, the inflation-adjusted value of state-level excise taxes was significantly associated with RIP. Over the study period, every additional dollar levied on cigarette excise taxes at the state-level was associated with a 9% increase in RIP. In other words, for two demographically similar smokers living in states where excise taxes differed by one dollar, the smoker living in the higher-taxed state spent, on average, 9% more of his/her annual per capita household income on cigarettes (*p* < 0.001). This was true even after controlling for differences in the labor force participation rate between states.

## 4. Discussion

In general, there was greater variation in self-reported cigarette prices across US states from 2003 to 2015 than there was in the affordability of cigarettes. Cigarette affordability considers the prices smokers pay for cigarettes in relation to their incomes. While average self-reported prices were substantially higher in some states compared to others, once smokers’ incomes were considered, smokers living in different states could have had similar levels of cigarette affordability. For example, smokers from Connecticut reported paying $8.50/pack in 2015 (average real price) while smokers from Michigan only paid $6.12/pack. While state excise taxes may largely explain this difference, once smokers’ incomes were considered, cigarettes were equally affordable in either state. In either state, smokers spent about 4.2% of their annual per capita household incomes to purchase 100 standard packs of 20 cigarettes. Thus, one way to assess the possible effects of tax-induced cigarette price increases is to determine whether such increases make cigarettes substantially less affordable for smokers.

While the self-reported real price of a pack of 20 cigarettes increased across all states over the course of the study, cigarettes only became slightly less affordable in most states from 2003 to 2008, as indicated by a 3.4% annual increase in RIP during this time period. While Bandi et al. [16] noted that cigarettes became slightly more affordable from 2001 to 2008, that trend was not statistically significant. Moreover, Bandi et al. [16] used an aggregate measure of relative income price to examine trends in affordability while this study used individual-level data. As a result, estimates between these studies are not directly comparable. In spite of that, both studies would support the notion that cigarettes remained relatively affordable for US smokers during this time period.

These results also demonstrate that cigarettes became substantially less affordable after the federal cigarette excise tax was increased in 2009. Across all US states, cigarette RIP increased by 30% from 2008 to 2010 meaning that cigarettes became less affordable for US smokers. While the effects of the tax increase cannot be disentangled from the effects of the economic recession, the longitudinal nature of the data made it possible to assess whether the recession had any lingering effects on cigarette affordability. Indeed, cigarettes continued to become less affordable for US smokers from 2010 to 2013. Since 2013, however, cigarettes become more affordable for US smokers, likely as a result of improved economic conditions.

Over and above these trends, state-level excise taxes were associated with significantly reduced cigarette affordability over the course of the study. Specifically, a $1 increase in the inflation-adjusted state-level cigarette tax was associated with a 9% increase in RIP, meaning that state-level excise taxes were associated with decreased cigarette affordability for US smokers. Taken as a whole, these results demonstrate that both federal and state-level cigarette excise taxes are associated with significantly reduced cigarette affordability for individual smokers.

### Strengths and Limitations

Unlike previous studies relying on aggregate affordability measures, individual-level affordability estimates from this study provide a better reflection of smokers’ actual ability to pay for cigarettes. The longitudinal study design made it possible to test whether the federal tax increase of 2009 was significantly associated with reduced affordability, even though the effect of the tax increase could not be disentangled from underlying economic conditions. The longitudinal design also made it possible to examine changes in affordability in subsequent time periods. In addition, small area estimation models were used to estimate individual-level affordability across all 50 states and the District of Columbia. Similar trends in affordability were observed across most states. Consistent with the overall national trend, state-level trends suggest the federal tax increase was at least partially responsible for reduced cigarette affordability in most states following its implementation in 2009.

In spite of these strengths, some limitations must be considered. First, the decrease in RIP (i.e., increased affordability) from 2013 to 2015 may be an artifact of splitting the last wave of data into two sub-waves. In particular, in Wave 9 of the ITC US survey, the majority of new replenishment respondents were surveyed in 2015. Thus, the increase in affordability observed by 2015 may have been attributable to this new sample of smokers. To test whether this might be the case, an additional LME model was estimated including time-in-sample as a covariate in the model. In this model, time-in-sample was assigned a value of 1 for respondents’ first wave of appearance and a value of 2 for an appearance in all subsequent waves. Differences between parameter estimates for this model and the four-slope model presented in Table 3 were negligible. Moreover, the time-in-sample covariate was not statistically significant.

Furthermore, the increase in affordability from 2013 to 2015 is consistent with data suggesting the US economy had fully recovered by this time. For example, while the recession officially ended in June 2009, the national unemployment rate peaked at 10% in October 2009. By November 2010, the unemployment rate began a steady decline until it reached pre-recession levels in September 2015 [32]. Median household income had also returned to pre-recession levels by 2015 [33]. Unlike these economic indicators, however, cigarette affordability had not dropped to pre-tax, pre-recession levels by the end of the study. Thus, increased excise taxes may have helped to reduce affordability in the long-term, although it is clear from these results that cigarette affordability is closely tied to prevailing economic conditions.

A second limitation to consider is the use of imputed per capita household income to compute cigarette affordability. Because the ITC survey collected income in eight broad categories, it was necessary to impute a continuous measure of per capita household income in order to compute cigarette RIP. Previous research used a similar approach to estimate cigarette affordability in Bangladesh [11]. Moreover, the distribution of imputed incomes was similar across all ITC survey waves (Figure S2) suggesting the imputation procedure was able to assign per capita income consistently across survey waves.

Third, while segmented LME models controlled for state-level taxes, they did not account for local taxes across jurisdictions within states. Thus, the overall effect of state-level taxes may be biased. The direction and magnitude of that bias would depend on the proportion of respondents living in jurisdictions having economically meaningful local taxes.

Finally, the analysis did not account for smokers who try to avoid federal and/or state excise taxes by purchasing cigarettes out-of-state, from duty-free shops, from Indian reservations, or from military commissaries. Smokers purchasing such low or untaxed sources of cigarettes lower their overall costs making cigarettes more affordable for them. These types of purchases would reduce estimates of RIP. Overall, only 4.1–6.3% of smokers in any given survey wave made such purchases. The low prevalence of low/untaxed purchases would exert only minimal effects on estimated affordability. Furthermore, since the proportion of such purchases was relatively constant across waves, the observed trends should not be affected in any systematic fashion.

## 5. Conclusions

While an earlier study estimated temporal trends in cigarette affordability across all US states using aggregate measures of affordability [16], this study is the first to our knowledge to estimate state- and national-level trends in affordability using individual-level data from the US. The current study illustrates that both federal- and state-level cigarette tax increases decrease cigarette affordability, or the price smokers pay for cigarettes in relation to their incomes. Underlying economic conditions further influence affordability via income. Affordability measures, such as relative income price, should be adopted to assess the effects of tax increases on smokers’ ability to pay for cigarettes. Reducing affordability ultimately influences smokers’ decisions to quit smoking, to reduce their consumption, or to actively search for lower-cost sources of cigarettes. Tax policies that are not responsive to underlying economic conditions ultimately make cigarettes more affordable for smokers. Tax-induced price increases must therefore keep pace with inflationary pressures to ensure cigarettes do not become more affordable over time. While taxes play an important role in reducing cigarette affordability, it is also necessary to understand smokers’ behavioral reactions to changes in affordability. For example, does reduced affordability lead smokers to quit smoking completely, to continue smoking but reduce their consumption, or to find cheaper sources of cigarettes? While these critically important questions are beyond the scope of this study, existing research demonstrates that some smokers do indeed quit in the face of tax increases [35,36,37,38] while others rely on price-minimization strategies [39].

## Figures and Tables

**Table 1:** Data sources used for state-level estimation of cigarette affordability.

International Tobacco Control US Survey	Survey Start/End	American Community Survey	Behavioral Risk Factor Surveillance System
Wave 2	May 2003	2003	2003
	September 2003		
Wave 3	June 2004	2004	2004
	December 2004		
Wave 4	October 2005	2005	2005
	January 2006	2006	
Wave 5	October 2006	2006	2006
	February 2007	2007	
Wave 6	September 2007	2007	2007
	February 2008	2008	
Wave 7	October 2008	2008	2008
	July 2009	2009	
Wave 8	July 2010	2010	2010
	June 2011	2011	
Wave 9			
9a	August 2013	2013	2013
	October 2014	2014	
9b	February 2015	2015	2015
	April 2015		

**Table 2:** Characteristics of smokers by wave of recruitment into the ITC US Survey.

Characteristic	Wave 1 (2002)	Wave 2 (2003)	Wave 3 (2004)	Wave 4 (2005)	Wave 5 (2006)	Wave 6 (2007)	Wave 7 (2008)	Wave 8 (2010)	Wave 9 (2013)
	(*n* = 1214)	(*n* = 655)	(*n* = 855)	(*n* = 710)	(*n* = 710)	(*n* = 666)	(*n* = 354)	(*n* = 342)	(*n* = 1540)
Mean time-in-sample (SD)	3.63 (2.06)	2.41 (1.91)	2.31 (1.76)	2.15 (1.55)	2.13 (1.40)	2.08 (1.11)	1.63 (0.78)	1.32 (0.47)	1.00 (0.00)
Male (%)	501 (41.3)	310 (47.3)	353 (41.3)	288 (40.6)	292 (41.1)	295 (44.3)	222 (62.7)	193 (56.4)	766 (49.7)
Age group (%)
18–24	132 (10.9)	94 (14.4)	98 (11.5)	75 (10.6)	56 (7.9)	36 (5.4)	19 (5.4)	12 (3.5)	92 (6.0)
25–39	319 (26.3)	171 (26.1)	237 (27.7)	197 (27.7)	156 (22.0)	107 (16.1)	68 (19.2)	53 (15.5)	346 (22.5)
40–54	463 (38.1)	239 (36.5)	318 (37.2)	241 (33.9)	303 (42.7)	264 (39.6)	146 (41.2)	125 (36.5)	452 (29.4)
55+	300 (24.7)	151 (23.1)	202 (23.6)	197 (27.7)	195 (27.5)	259 (38.9)	121 (34.2)	152 (44.4)	650 (42.2)
≤High school education (%)	496 (40.9)	245 (37.4)	403 (47.1)	357 (50.3)	368 (51.8)	304 (45.6)	165 (46.6)	142 (41.5)	623 (40.5)
Income (%) *
low	421 (34.7)	250 (38.2)	310 (36.3)	272 (38.3)	271 (38.2)	209 (31.4)	123 (34.7)	129 (37.7)	592 (38.4)
moderate	445 (36.7)	220 (33.6)	317 (37.1)	228 (32.1)	225 (31.7)	218 (32.7)	97 (27.4)	91 (26.6)	439 (28.5)
high	272 (22.4)	148 (22.6)	191 (22.3)	169 (23.8)	169 (23.8)	191 (28.7)	87 (24.6)	85 (24.9)	502 (32.6)
not reported	76 (6.3)	37 (5.6)	37 (4.3)	41 (5.8)	45 (6.3)	48 (7.2)	47 (13.3)	37 (10.8)	7 (0.5)
Ages of children in home (%)
children under 6 only ^†^	99 (8.2)	68 (10.4)	78 (9.1)	55 (7.8)	65 (9.2)	30 (4.5)	17 (4.8)	17 (5.0)	82 (5.3)
children 6 to 17 only ^†^	268 (22.2)	152 (23.3)	179 (20.9)	144 (20.3)	147 (20.7)	122 (18.3)	71 (20.2)	47 (13.9)	273 (17.7)
both	115 (9.5)	45 (6.9)	93 (10.9)	65 (9.2)	55 (7.8)	27 (4.1)	25 (7.1)	15 (4.4)	81 (5.3)
no children	723 (60.0)	387 (59.4)	505 (59.1)	445 (62.8)	442 (62.3)	487 (73.1)	239 (67.9)	260 (76.7)	1104 (71.7)
Race/ethnicity (%)
White	965 (79.5)	495 (75.6)	699 (81.8)	561 (79.0)	567 (79.9)	553 (83.0)	258 (72.9)	247 (72.2)	1131 (73.4)
Black	103 (8.5)	77 (11.8)	66 (7.7)	61 (8.6)	77 (10.8)	58 (8.7)	39 (11.0)	40 (11.7)	168 (10.9)
Hispanic	57 (4.7)	36 (5.5)	35 (4.1)	26 (3.7)	31 (4.4)	15 (2.3)	12 (3.4)	13 (3.8)	135 (8.8)
other	89 (7.3)	47 (7.2)	55 (6.4)	62 (8.7)	35 (4.9)	40 (6.0)	45 (12.7)	42 (12.3)	106 (6.9)
Employed (%)	798 (65.7)	414 (63.2)	517 (60.5)	401 (56.5)	383 (53.9)	340 (51.1)	179 (50.6)	161 (47.1)	771 (50.1)
Daily smoker (%)	1103 (91.0)	598 (91.3)	799 (93.5)	671 (94.5)	684 (96.3)	634 (95.2)	325 (91.8)	312 (91.2)	1280 (83.1)
Mean cigarettes/day (SD)	17.06 (10.78)	17.89 (11.02)	17.85 (10.76)	18.59 (11.66)	19.67 (12.02)	19.63 (11.47)	16.63 (10.79)	16.61 (11.12)	12.91 (9.90)
Last purchased cigarette packs (%)	699 (57.6)	394 (60.2)	517 (60.5)	413 (58.2)	398 (56.1)	344 (51.7)	235 (66.4)	213 (62.3)	1091 (70.8)

* Here, income refers to self-reported income as collected in the ITC US Survey, not the imputed version of income. ^†^ In Waves 8 and 9, these two levels were classified as “children under 5 only” and “children 5 to 17”, respectively, due to changes in the American Community Survey data used to impute per capita household income.

**Table 3:** Comparison of temporal trends in log(relative income price) across the United States, 2003–2015 (*n* = 12,322 observations).

	1-Slope Model		2-Slope Model		3-Slope Model		4-Slope Model	
	β	(SE)	β	(SE)	β	(SE)	β	(SE)
**Fixed Effects**	
(Intercept)	−1.753	(0.293) ^†^	−3.000	(0.294) ^†^	−3.087	(0.299) ^†^	−3.184	(0.298) ^†^
Gender (female vs. male)	0.206	(0.021) ^†^	0.210	(0.021) ^†^	0.210	(0.021) ^†^	0.207	(0.021) ^†^
Age group (25–39 vs. 18–24)	−0.056	(0.041)	−0.057	(0.041)	−0.054	(0.041)	−0.053	(0.041)
Age group (40–54 vs. 18–24)	−0.331	(0.040) ^†^	−0.326	(0.040) ^†^	−0.328	(0.040) ^†^	−0.328	(0.040) ^†^
Age group (55+ vs. 18–24)	−0.444	(0.041) ^†^	−0.446	(0.041) ^†^	−0.439	(0.041) ^†^	−0.435	(0.041) ^†^
Race/ethnicity (Black vs. white)	0.672	(0.035) ^†^	0.667	(0.035) ^†^	0.669	(0.035) ^†^	0.671	(0.035) ^†^
Race/ethnicity (Hispanic vs. white)	0.320	(0.049) ^†^	0.301	(0.049) ^†^	0.322	(0.048) ^†^	0.334	(0.049) ^†^
Race/ethnicity (other vs. white)	0.216	(0.040) ^†^	0.215	(0.040) ^†^	0.210	(0.040) ^†^	0.211	(0.040) ^†^
High school education (vs greater)	0.322	(0.020) ^†^	0.326	(0.020) ^†^	0.325	(0.020) ^†^	0.324	(0.020) ^†^
Employed (vs otherwise)	−0.199	(0.014) ^†^	−0.199	(0.014) ^†^	−0.193	(0.014) ^†^	−0.190	(0.014) ^†^
Surveyed in post-tax period of 2009	0.035	(0.039)	0.101	(0.040) ^‡^	0.173	(0.041) ^†^	0.174	(0.041) ^†^
State excise tax (in 2015 USD)	0.108	(0.013) ^†^	0.106	(0.012) ^†^	0.089	(0.012) ^†^	0.089	(0.012) ^†^
Labour force participation rate	−0.032	(0.004) ^†^	−0.014	(0.004) ^‡^	−0.012	(0.004) ^‡^	−0.010	(0.004) ^§^
Period 1 *	0.051	(0.003) ^†^	0.037	(0.004) ^†^	0.032	(0.004) ^†^	0.033	(0.004) ^†^
Period 2 *			0.078	(0.011) ^†^	0.240	(0.020) ^†^	0.230	(0.020) ^†^
Period 3 *					−0.268	(0.027) ^†^	−0.193	(0.034) ^†^
Period 4 *							−0.173	(0.047) ^†^
**Random Effects**	
N respondents	6660		6660		6660		6660	
N state	51		51		51		51	
$\sigma^{2}$	0.1418		0.1409		0.1392		0.1400	
$\tau_{00}$, state:respondent	0.5932		0.5952		0.5927		0.5926	
$\tau_{00}$, state	0.0204		0.0102		0.0112		0.0110	
ICC state:respondent	0.7853		0.7976		0.7975		0.7980	
ICC state	0.0270		0.0136		0.0151		0.0148	
AIC	24,381.02		24,335.52		24,238.74		24,227.14	
Test of random effects ($\chi^{2}$)	
state:respondent	6098.3 ^†^		6108.1 ^†^		6157.8 ^†^		6168.6 ^†^	
state	46.0 ^†^		28.3 ^†^		31.6 ^†^		31.1 ^†^	

In each model, “period” refers to different effects depending on the number of slopes estimated. In the single-slope model, $\beta_{\mathrm{period}\;1}$ estimates the wave-to-wave rate of change in log(RIP) over the entire study period from 2003 to 2015. In the two-slope model, $\beta_{\mathrm{period}\;1}$ estimates the wave-to-wave rate of change in log(RIP) from 2003 to 2008 while $\beta_{\mathrm{period}\;2}$ estimates the difference between slopes for Period 1 compared to Period 2 (2008 to 2015). In the three-slope model, $\beta_{\mathrm{period}\;1}$ refers to the same period effect as model 2 while $\beta_{\mathrm{period}\;2}$ estimates the difference between slopes from 2008 to 2010 compared to 2003 to 2008. $\beta_{\mathrm{period}\;3}$ estimates the difference between the slope for 2010 to 2015 compared to the previous two time periods. Finally, in the four-slope model $\beta_{\mathrm{period}\;1}$ and $\beta_{\mathrm{period}\;2}$ refer to the same effects as in Model 3, while $\beta_{\mathrm{period}\;3}$ estimates the difference between the slope from 2010 to 2013 compared to the previous two periods. $\beta_{\mathrm{period}\;4}$ estimates the difference between the slope from 2013 to 2015 compared to the previous three periods. ^†^ *p* < 0.001; ^‡^ *p* < 0.01; ^§^ *p* < 0.05.

## Supplementary Materials

The following are available online at https://www.mdpi.com/1660-4601/16/13/2439/s1, Figure S1: Process used to impute a continuous measure of income for smokers participating in the ITC US Survey (Waves 2–9) using proxy data from a contemporaneous American Community Survey. Figure S2: Distribution of imputed per capita household income for ITC US respondents by survey wave. Figure S3: Inflation adjusted self-reported cigarette prices (per standard pack of 20 cigarettes in 2015 USD) across all US states from 2003 to 2015. Figure S4: Cigarette affordability, measured by the relative income price (RIP) of cigarettes, across all US states from 2003 to 2015. Higher values of RIP, represented by darker shades, indicate lower levels of affordability. Table S1: Wave-specific linear mixed effects models to estimate state-level real cigarette prices per pack of 20 in 2015 USD (ITC US Survey Wave 2–Wave 9). Table S2: Self-reported real cigarette price/pack of 20 (2015 USD) by state from 2003 to 2015 (ITC US Survey Wave 2–Wave 9). Table S3: Wave-specific linear mixed effects models to estimate state-level cigarette affordability (log(RIP), ITC US Survey Wave 2–Wave 9). Table S4: Cigarette affordability (relative income price) across all US states from 2003 to 2015 (ITC US Survey Wave 2–Wave 9).

## Abbreviations

The following abbreviations are used in this manuscript:

ITC	International Tobacco Control
US	United States
RIP	relative income price
ACS	American Community Survey
BRFSS	Behavioral Risk Factor Surveillance System
CATI	computer-assisted telephone interviewing
LME	linear mixed effects
EBLUP	empirical best linear unbiased predictor
ICC	intraclass correlation
AIC	Akaike’s Information Criteria statistic
