# Supplementary material for: State-Level Affordability of Factory-Made Cigarettes among Current US Smokers: Findings from the ITC US Survey, 2003–2015
Source: Int J Environ Res Public Health. 2019 Jul 9;16(13):2439. doi: 10.3390/ijerph16132439 (PMC6650842; doi:10.3390/ijerph16132439)
Supplement: Supplementary file 1 [file ijerph-16-02439-s001.zip › affordability-supplementary-figures.pdf]

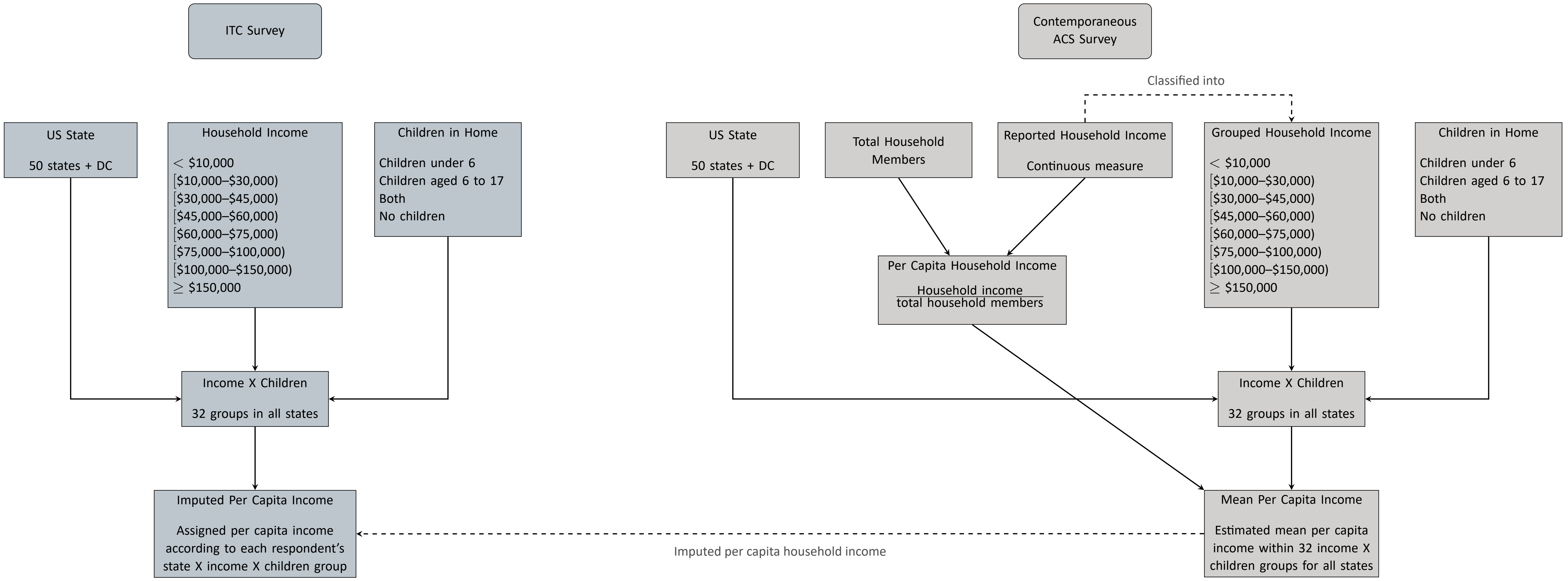

Figure S1. Process used to impute a continuous measure of income for smokers participating in the ITC US Survey (Waves 2–9) using proxy data from a contemporaneous American Community Survey.

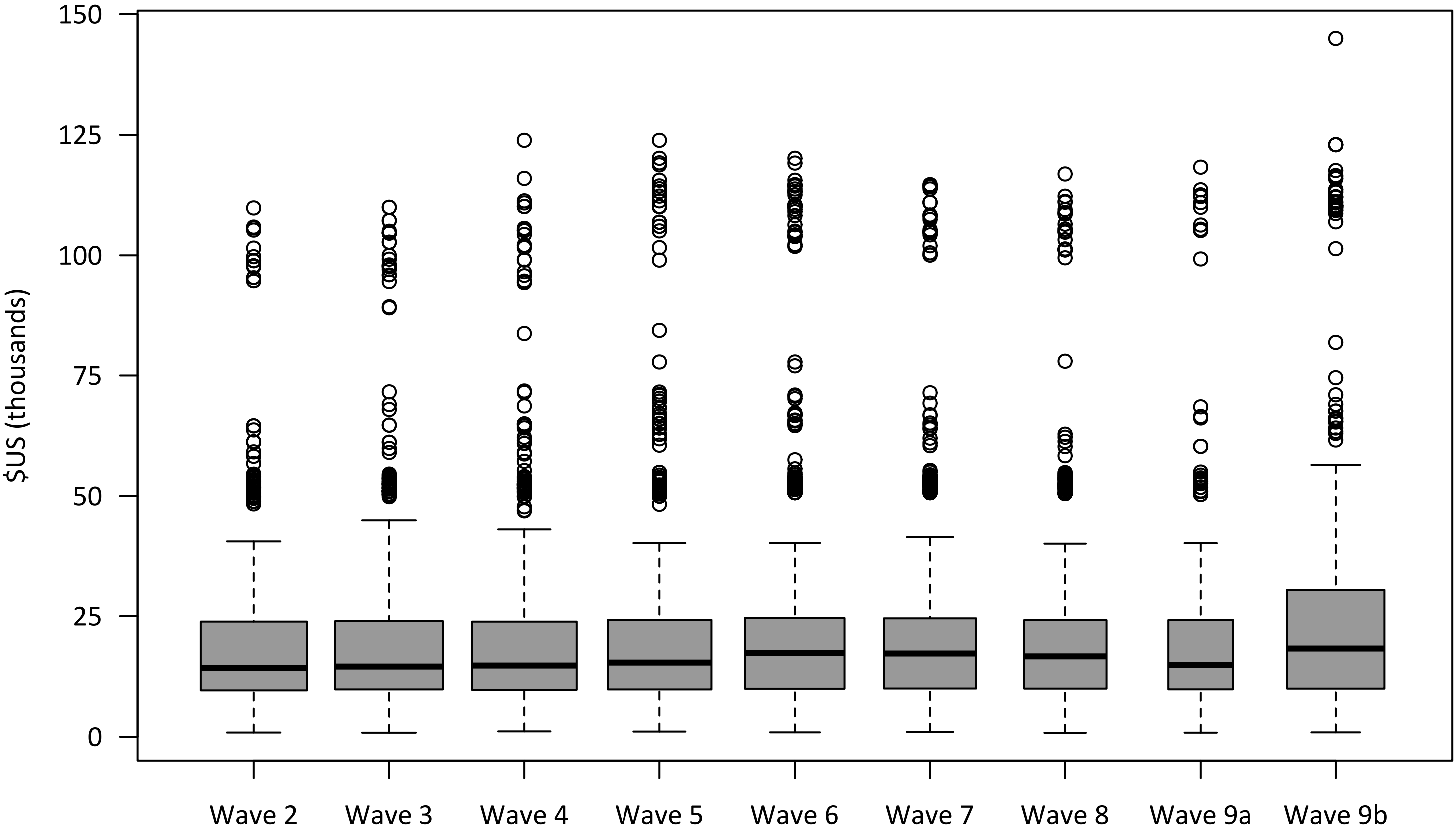

Figure S2. Distribution of imputed per capita household income for ITC US respondents by survey wave.

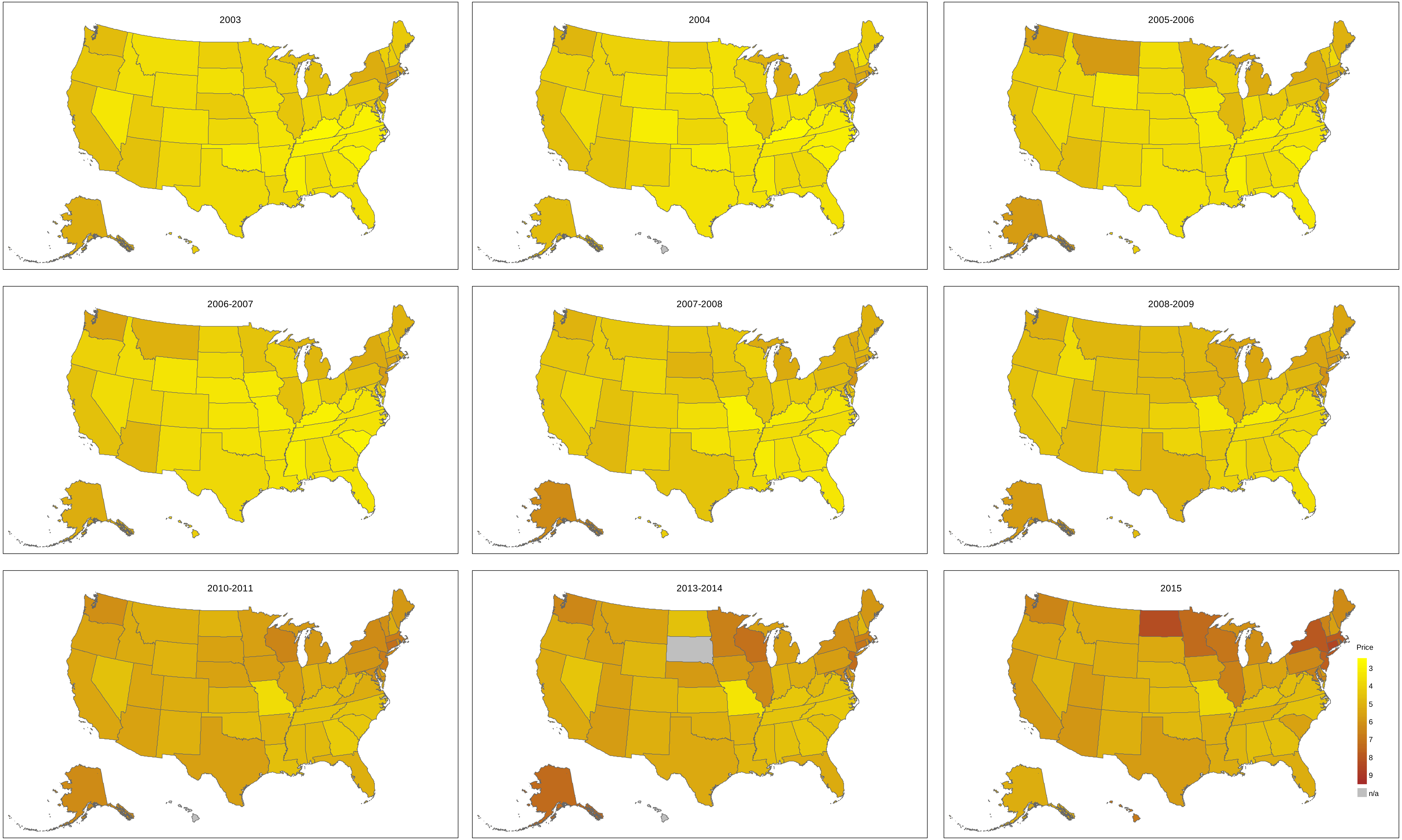

Figure S3. Inflation adjusted self-reported cigarette prices (per standard pack of 20 cigarettes in 2015 USD) across all US states from 2003 to 2015.

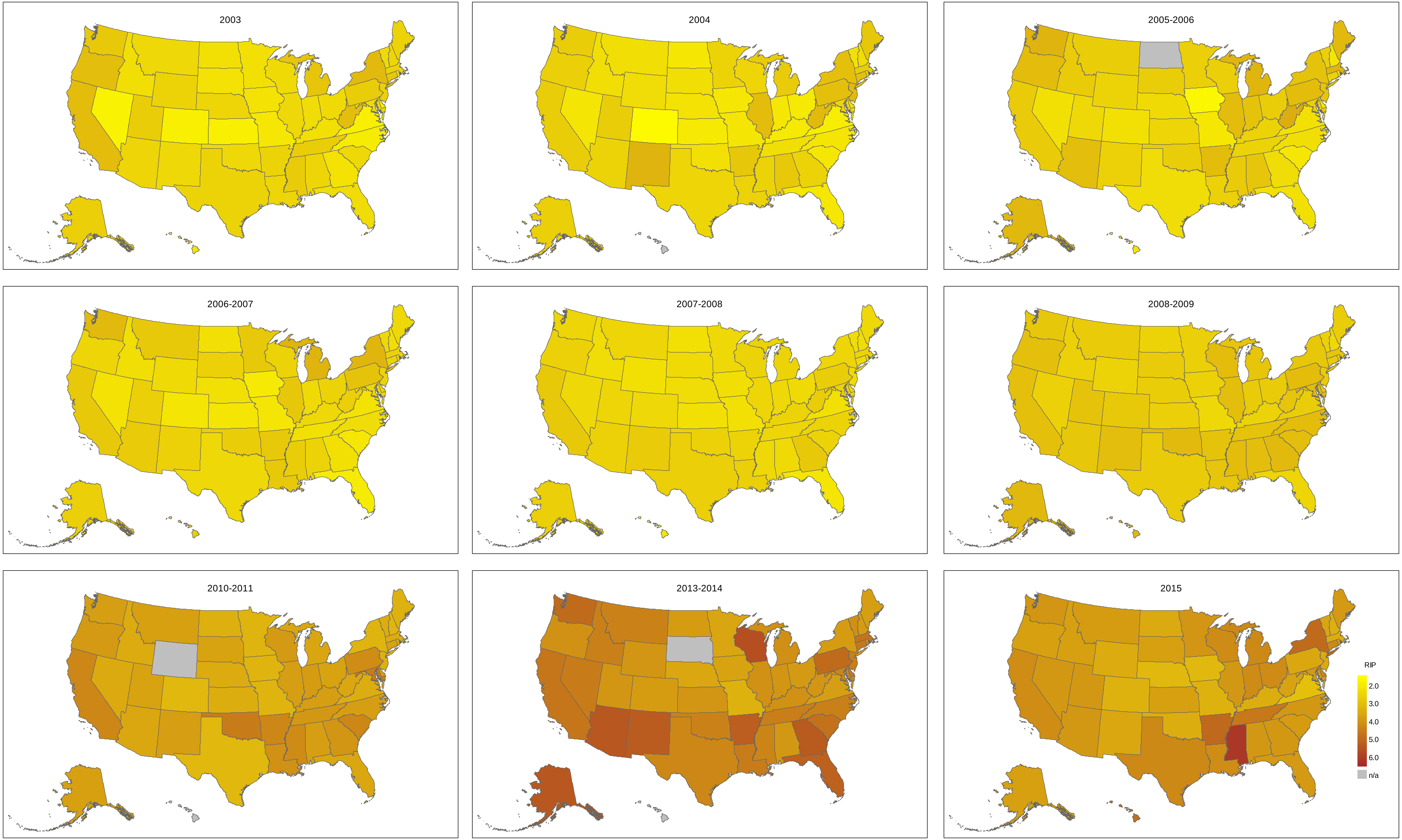

Figure S4. Cigarette affordability, measured by the relative income price (RIP) of cigarettes, across all US states from 2003 to 2015. Higher values of RIP, represented by darker shades, indicate lower levels of affordability.
