# Supplementary material for: State-Level Affordability of Factory-Made Cigarettes among Current US Smokers: Findings from the ITC US Survey, 2003–2015
Source: Int J Environ Res Public Health. 2019 Jul 9;16(13):2439. doi: 10.3390/ijerph16132439 (PMC6650842; doi:10.3390/ijerph16132439)
Supplement: Supplementary file 1 [file ijerph-16-02439-s001.zip › affordability-supplementary-tables.pdf]

Table S1. Wave-specific linear mixed effects models to estimate state-level real cigarette prices per pack of 20 in 2015 USD (ITC US Survey Wave 2–Wave 9).

	Wave 2 (2003) n = 1805	Wave 3 (2004) n = 1835	Wave 4 (2005) n = 1720	Wave 5 (2006) n = 1697	Wave 6 (2007) n = 1573	Wave 7 (2008) n = 1366	Wave 8 (2010) n = 1124	Wave 9a (2013) n = 668	Wave 9b (2015) n = 1403
	β (SE)	β (SE)	β (SE)	β (SE)	β (SE)	β (SE)	β (SE)	β (SE)	β (SE)
**Fixed effects**									
(Intercept)	4.32 (0.15)*	4.13 (0.16)*	4.25 (0.17)*	4.14 (0.18)*	4.51 (0.20)*	5.39 (0.25)*	5.83 (0.32)*	5.20 (0.45)*	5.55 (0.28)*
Gender (female vs male)	-0.09 (0.06)	-0.01 (0.06)	0.02 (0.06)	0.02 (0.06)	0.00 (0.06)	-0.11 (0.08)	-0.18 (0.10)	0.16 (0.13)	-0.04 (0.10)
Age group (25-39 vs 18-24)	-0.32 (0.10)†	-0.14 (0.11)	-0.15 (0.12)	-0.14 (0.13)	-0.20 (0.17)	-0.77 (0.23)*	-0.49 (0.30)	0.28 (0.44)	0.41 (0.22)
Age group (40-54 vs 18-24)	-0.60 (0.09)*	-0.51 (0.10)*	-0.55 (0.11)*	-0.47 (0.12)*	-0.51 (0.16)†	-1.17 (0.22)*	-0.53 (0.28)	-0.04 (0.41)	-0.05 (0.21)
Age group (55+ vs 18-24)	-0.80 (0.10)*	-0.71 (0.11)*	-0.71 (0.12)*	-0.70 (0.13)*	-0.72 (0.16)*	-1.37 (0.22)*	-0.75 (0.29)†	-0.21 (0.41)	-0.31 (0.21)
Race/ethnicity (Black vs white)	0.60 (0.10)*	0.84 (0.11)*	0.81 (0.11)*	0.54 (0.11)*	0.61 (0.12)*	0.81 (0.14)*	1.00 (0.17)*	0.69 (0.24)†	0.71 (0.16)*
Race/ethnicity (Hispanic vs white)	0.43 (0.13)†	0.42 (0.15)†	0.09 (0.18)	0.35 (0.16)‡	0.35 (0.19)	0.21 (0.25)	0.82 (0.33)‡	0.13 (0.60)	0.92 (0.17)*
Race/ethnicity (other vs white)	0.02 (0.11)	0.11 (0.12)	0.38 (0.11)†	0.32 (0.13)‡	0.27 (0.14)‡	0.28 (0.16)	-0.07 (0.19)	0.14 (0.29)	0.30 (0.20)
High school education (vs greater)	-0.12 (0.06)‡	-0.12 (0.06)‡	0.00 (0.06)	-0.06 (0.06)	-0.11 (0.06)	-0.20 (0.08)†	-0.32 (0.10)†	-0.34 (0.13)‡	-0.26 (0.10)‡
Employed (vs otherwise)	0.20 (0.06)†	0.20 (0.06)†	0.19 (0.06)†	0.26 (0.06)*	0.19 (0.07)†	0.26 (0.08)†	0.25 (0.10)‡	0.35 (0.14)‡	0.30 (0.10)†
**Random effects**									
N state	51	51	51	51	50	50	49	49	51
σ^2	1.381	1.403	1.328	1.364	1.434	1.875	2.512	2.669	3.143
τ_{00}, state	0.573	0.653	0.699	0.640	0.612	0.579	0.697	1.001	1.352
ICC state	0.293	0.318	0.345	0.319	0.299	0.236	0.217	0.273	0.301
Test of random effect: χ^2_1	455*	560*	525*	448*	318*	229*	145*	103*	297*

* $p < 0.001$; † $p < 0.01$; ‡ $p < 0.05$

Table S2. Self-reported real cigarette price/pack of 20 (2015 USD) by state from 2003 to 2015 (ITC US Survey Wave 2–Wave 9).

Region	State	Wave 2 (2003)	Wave 3 (2004)	Wave 4 (2005)	Wave 5 (2006)	Wave 6 (2007)	Wave 7 (2008)	Wave 8 (2010)	Wave 9a (2013)	Wave 9b (2015)	Trend
Northeast	CT	5.70	5.29	5.07	5.38	5.46	5.85	7.18	6.96	8.50	
	MA	5.38	5.00	5.15	4.96	4.88	5.76	6.72	6.64	7.86	
	ME	4.31	4.23	5.05	4.94	5.07	5.39	5.88	5.98	6.28	
	NH	4.14	3.61	3.82	4.08	4.63	4.39	5.24	4.91	5.63	
	NJ	5.69	6.32	5.80	5.76	5.94	6.05	6.77	7.28	7.67	
	NY	5.18	5.07	5.27	5.23	4.96	5.40	6.27	6.04	7.88	
	PA	4.25	4.61	4.50	4.62	4.64	4.88	5.93	5.67	6.33	
	RI	4.28	5.81	4.78	5.34	5.27	5.18	6.05	6.13	7.25	
	VT	4.11	3.77	4.42	4.52	5.37	5.03	6.11	5.83	6.55	
Midwest	IA	3.48	3.27	3.19	3.28	4.51	5.07	5.69	5.81	5.54	
	IL	4.41	4.55	4.84	4.58	4.52	5.11	5.62	6.34	6.64	
	IN	3.87	3.59	3.71	3.52	4.23	4.59	4.98	4.91	5.35	
	KS	3.80	3.93	3.61	3.36	3.63	4.05	4.84	4.60	4.67	
	MI	4.58	5.04	5.25	4.90	5.22	5.38	5.90	5.60	6.12	
	MN	4.04	3.57	4.96	4.48	4.44	4.83	5.61	6.60	7.31	
	MO	3.40	3.06	3.21	3.07	3.00	3.27	3.67	3.42	3.82	
	ND	4.07	4.16	3.70	4.06	4.32	4.68	4.99	4.57	8.25	
	NE	4.21	3.75	3.73	3.32	4.33	4.75	5.52	5.83	4.92	
	OH	3.80	3.56	4.28	4.27	4.30	4.70	5.26	5.19	5.46	
	SD	3.54	3.34	3.84	3.72	4.95	4.75	5.54	n/a	5.34	
	WI	4.08	3.96	4.02	4.03	4.12	5.30	6.45	7.09	6.99	
South	AL	3.66	3.85	3.79	3.68	3.62	4.16	4.72	4.52	4.60	
	AR	3.35	3.54	3.85	3.52	3.84	4.38	4.94	5.00	5.18	
	DC	5.53	4.85	4.78	4.54	n/a	n/a	n/a	5.18	6.36	
	DE	3.43	3.42	3.57	3.70	4.41	4.57	5.39	5.50	5.82	
	FL	3.55	3.48	3.28	3.42	3.29	3.55	5.13	5.24	5.16	
	GA	3.35	3.83	3.57	3.37	3.45	3.96	4.21	4.37	4.57	
	KY	2.76	2.68	3.03	2.97	3.17	3.17	4.59	4.34	4.45	
	LA	3.87	3.48	3.66	3.45	3.75	4.03	4.59	4.87	5.22	
	MD	4.32	4.30	4.26	4.16	4.63	5.56	6.12	6.19	6.52	
	MS	3.05	3.15	3.02	3.10	3.18	3.67	4.74	4.70	4.87	
	NC	3.11	3.05	3.33	3.43	3.67	3.93	4.45	4.34	4.54	
	OK	3.14	3.12	3.66	3.49	3.44	3.95	4.68	5.09	4.82	
	SC	2.90	2.95	2.99	2.86	3.07	3.50	4.33	4.61	5.56	
	TN	3.06	3.35	3.39	3.17	3.71	3.79	4.48	4.44	5.19	
	TX	3.75	3.45	3.49	3.82	4.48	4.96	5.62	5.33	5.74	
	VA	3.00	3.17	3.39	3.49	3.39	3.86	5.16	4.48	4.74	
	WV	3.32	3.10	3.30	3.29	3.63	3.98	4.78	4.86	4.70	
West	AK	5.23	4.74	5.97	5.21	6.04	5.52	6.32	7.27	5.39	
	AZ	4.51	4.49	4.65	4.92	4.85	4.85	5.51	5.79	5.97	
	CA	4.70	4.64	4.40	4.55	4.37	4.55	5.37	5.29	5.82	
	CO	3.51	3.13	3.85	3.81	4.11	4.44	5.17	4.89	5.06	
	HI	4.30	n/a	4.42	4.40	4.42	4.80	n/a	n/a	6.74	
	ID	3.58	3.95	3.81	3.65	4.13	3.66	5.24	5.60	4.97	
	MT	3.59	4.01	5.83	5.02	4.36	4.87	5.18	5.57	5.35	
	NM	3.97	4.04	3.95	3.71	4.05	4.16	5.04	5.14	5.12	
	NV	3.29	3.68	3.78	3.66	3.84	4.06	4.55	4.40	4.90	
	OR	4.32	4.04	4.14	4.00	4.43	4.65	5.44	5.18	5.33	
	UT	4.19	4.20	3.98	4.02	4.53	4.84	5.40	5.63	5.74	
	WA	4.70	4.91	5.51	5.44	4.96	5.12	6.09	6.40	6.46	
	WY	3.69	3.46	3.36	3.37	3.85	4.51	4.95	4.88	5.23	

n/a: No observations in a given state and survey wave to estimate self-reported price.

Table S3. Wave-specific linear mixed effects models to estimate state-level cigarette affordability (log(RIP), ITC US Survey Wave 2–Wave 9).

	Wave 2 (2003) n = 1691	Wave 3 (2004) n = 1740	Wave 4 (2005) n = 1623	Wave 5 (2006) n = 1605	Wave 6 (2007) n = 1484	Wave 7 (2008) n = 1263	Wave 8 (2010) n = 1017	Wave 9a (2013) n = 619	Wave 9b (2015) n = 1403
	β (SE)	β (SE)	β (SE)	β (SE)	β (SE)	β (SE)	β (SE)	β (SE)	β (SE)
**Fixed effects**									
(Intercept)	-3.606 (0.080) *	-3.657 (0.083) *	-3.459 (0.096) *	-3.682 (0.104) *	-3.629 (0.116) *	-3.469 (0.137) *	-3.091 (0.180) *	-2.919 (0.240) *	-3.340 (0.116) *
Gender (female vs male)	0.197 (0.040) *	0.185 (0.039) *	0.145 (0.042) *	0.171 (0.043) *	0.241 (0.044) *	0.106 (0.046) ‡	0.000 (0.053)	0.163 (0.071) ‡	0.224 (0.049) *
Age group (25-39 vs 18-24)	-0.091 (0.069)	-0.094 (0.073)	-0.159 (0.083)	0.055 (0.095)	-0.030 (0.117)	-0.086 (0.140)	-0.176 (0.181)	-0.270 (0.246)	0.146 (0.110)
Age group (40-54 vs 18-24)	-0.412 (0.066) *	-0.387 (0.069) *	-0.470 (0.079) *	-0.298 (0.090) *	-0.315 (0.110) †	-0.369 (0.132) †	-0.417 (0.172) ‡	-0.533 (0.232) ‡	-0.075 (0.108)
Age group (55+ vs 18-24)	-0.555 (0.073) *	-0.523 (0.075) *	-0.581 (0.084) *	-0.380 (0.095) *	-0.421 (0.112) *	-0.437 (0.133) †	-0.487 (0.173) †	-0.588 (0.232) ‡	-0.396 (0.106) *
Race/ethnicity (Black vs white)	0.637 (0.069) *	0.767 (0.073) *	0.714 (0.079) *	0.500 (0.078) *	0.396 (0.079) *	0.634 (0.086) *	0.749 (0.093) *	0.569 (0.129) *	0.633 (0.080) *
Race/ethnicity (Hispanic vs white)	0.273 (0.094) †	0.440 (0.102) *	0.303 (0.125) ‡	0.352 (0.118) †	0.301 (0.132) ‡	0.180 (0.148)	0.153 (0.188)	0.639 (0.337)	0.263 (0.088) †
Race/ethnicity (other vs white)	0.185 (0.077) ‡	0.241 (0.083) †	0.231 (0.081) †	0.208 (0.094) ‡	0.092 (0.099)	0.395 (0.100) *	0.334 (0.106) †	0.175 (0.159)	0.285 (0.098) †
High school education (vs greater)	0.339 (0.041) *	0.314 (0.040) *	0.338 (0.042) *	0.309 (0.043) *	0.315 (0.044) *	0.336 (0.046) *	0.355 (0.054) *	0.293 (0.072) *	0.332 (0.051) *
Employed (vs otherwise)	-0.322 (0.045) *	-0.270 (0.042) *	-0.355 (0.044) *	-0.300 (0.046) *	-0.362 (0.046) *	-0.325 (0.048) *	-0.380 (0.056) *	-0.378 (0.077) *	-0.419 (0.052) *
**Random effects**									
N state	51	51	50	51	50	50	48	49	51
σ^2	0.6478	0.6222	0.6513	0.6824	0.6707	0.6410	0.6995	0.7333	0.8202
τ_{00}, state	0.0291	0.0378	0.0379	0.0264	0.0087	0.0096	0.0149	0.0341	0.0262
ICC state	0.0430	0.0572	0.0550	0.0373	0.0127	0.0148	0.0209	0.0444	0.0310
Test of random effect: χ^2_1	26.1*	38.4*	30.5*	23.5*	3.43	3.11	5.47‡	7.31†	8.65†

∗ $p < 0.001$; † $p < 0.01$; ‡ $p < 0.05$

Table S4. Cigarette affordability (relative income price) across all US states from 2003 to 2015 (ITC US Survey Wave 2–Wave 9).

Region	State	Wave 2 (2003)	Wave 3 (2004)	Wave 4 (2005)	Wave 5 (2006)	Wave 6 (2007)	Wave 7 (2008)	Wave 8 (2010)	Wave 9a (2013)	Wave 9b (2015)	Trend
Northeast	CT	2.82	2.97	2.53	2.54	2.37	2.73	3.67	4.65	4.23	
	MA	2.53	2.65	3.22	2.60	2.50	2.86	3.48	4.73	3.44	
	ME	2.54	2.81	3.11	2.42	2.43	2.68	3.35	3.79	3.86	
	NH	2.49	2.34	2.13	2.27	2.32	2.43	3.21	3.84	3.36	
	NJ	2.59	3.13	2.85	2.54	2.37	2.73	3.35	4.47	3.52	
	NY	3.11	3.00	2.92	3.21	2.47	2.80	3.28	3.81	4.93	
	PA	2.69	2.97	3.06	2.92	2.69	3.06	4.15	4.93	3.57	
	RI	2.17	2.48	2.40	2.48	2.35	2.66	3.40	4.11	3.74	
	VT	2.37	2.21	2.61	2.42	2.35	2.58	3.42	4.07	3.63	
Midwest	IA	2.16	2.07	1.71	2.00	2.27	2.60	3.27	3.67	3.16	
	IL	2.42	3.09	3.06	2.82	2.49	3.05	3.75	4.04	3.92	
	IN	2.33	2.09	2.90	2.38	2.44	2.74	3.82	3.98	4.17	
	KS	1.89	2.04	2.46	2.10	2.24	2.63	3.44	3.96	3.71	
	MI	2.89	2.73	3.21	3.26	2.53	2.86	3.64	4.09	4.20	
	MN	2.21	2.55	2.63	2.76	2.40	2.63	3.25	3.63	3.95	
	MO	2.07	2.11	2.05	2.13	2.23	2.44	3.28	3.31	3.34	
	ND	2.24	2.08	n/a	2.23	2.23	2.52	3.36	3.81	3.50	
	NE	2.49	2.19	2.39	2.24	2.32	2.69	3.48	3.57	3.19	
	OH	2.38	2.04	2.66	2.52	2.40	2.72	3.69	3.89	4.18	
	SD	2.19	2.28	2.68	2.55	2.37	2.70	3.68	n/a	3.71	
	WI	2.35	2.46	2.61	2.54	2.50	3.07	3.85	5.59	4.17	
South	AL	2.48	2.79	2.86	2.51	2.39	3.03	3.77	3.94	3.93	
	AR	2.55	2.81	3.08	2.76	2.52	2.92	4.28	5.22	4.95	
	DC	3.46	3.69	3.79	3.39	n/a	n/a	n/a	5.56	5.20	
	DE	2.03	2.16	2.06	2.46	2.41	2.77	3.71	4.57	4.27	
	FL	2.30	2.10	2.25	2.00	2.15	2.48	3.60	5.19	3.88	
	GA	2.19	2.52	2.32	2.25	2.76	3.10	3.98	5.33	3.94	
	KY	2.21	2.03	2.51	2.42	2.50	2.50	3.77	4.04	3.41	
	LA	2.46	2.53	2.57	2.80	2.61	2.87	4.04	4.50	4.12	
	MD	2.38	2.48	2.61	2.44	2.54	3.05	4.54	4.34	3.88	
	MS	2.75	2.53	2.71	2.67	2.44	3.05	4.20	4.34	6.11	
	NC	1.97	2.10	2.27	2.33	2.51	3.09	3.75	4.43	3.91	
	OK	2.42	2.22	2.66	2.66	2.59	3.15	4.51	4.39	3.44	
	SC	2.42	2.06	2.08	2.08	2.54	3.00	4.30	4.78	3.81	
	TN	2.68	2.29	2.54	2.21	2.49	2.94	3.92	4.46	4.61	
	TX	2.51	2.45	2.33	2.40	2.60	2.74	3.18	4.29	4.23	
	VA	1.86	1.91	2.21	2.16	2.21	2.69	3.45	3.76	2.98	
	WV	3.01	3.12	3.42	2.63	2.68	2.85	3.59	3.99	3.69	
West	AK	2.71	2.83	3.01	2.85	2.50	3.16	3.81	5.09	3.76	
	AZ	2.46	2.50	3.01	2.71	2.58	2.81	3.57	5.36	3.94	
	CA	3.05	2.70	2.72	2.75	2.75	2.97	4.28	4.66	4.10	
	CO	1.89	1.64	2.24	2.11	2.44	2.77	3.18	3.83	3.33	
	HI	2.40	n/a	2.20	2.52	2.43	3.27	n/a	n/a	4.61	
	ID	2.28	2.27	2.71	2.30	2.35	2.60	3.47	4.43	3.71	
	MT	2.44	2.27	2.66	2.76	2.49	2.75	3.75	4.39	3.85	
	NM	2.41	3.28	2.60	2.59	2.75	3.02	3.76	5.26	3.56	
	NV	1.83	2.15	2.25	2.16	2.42	2.59	3.50	4.51	3.90	
	OR	3.03	2.61	3.06	2.48	2.59	2.97	3.90	4.04	3.72	
	UT	2.66	2.68	2.42	2.64	2.50	2.90	3.68	3.98	3.93	
	WA	2.76	2.67	3.29	3.12	2.47	2.83	3.77	4.94	3.99	
	WY	2.53	2.31	2.50	2.39	2.23	2.52	n/a	3.96	3.49	

n/a: No observations in a given state and survey wave to estimate self-reported price.
